# Supplementary material for: Are carbon emissions trading and green financial instruments synergistic? -Comprehensive quantitative research based on content analysis
Source: PLoS One. 2024 Mar 7;19(3):e0298601. doi: 10.1371/journal.pone.0298601 (PMC10919601; doi:10.1371/journal.pone.0298601)
Supplement: S1 Data — (ZIP) [file pone.0298601.s001.zip › Data/地级市Z_F工作报告.zip!/─┌├╔╣┼/│α╖σ╩╨/└┤╫╘╣1⁄2╓┌║┼í╛┬φ┐╦╩2╛▌═°í┐_2012.docx]

各位代表：

　　现在，我代表市人民政府，向大会报告工作，请予审议，并请市政协委员和列席会议的同志提出意见。

　　一、2011年工作回顾

　　2011年是"十二五"开局之年，也是全市经济社会全面发展、重点工作实现较大突破的一年。在市委的正确领导下，全市上下深入贯彻落实科学发展观，牢牢把握发展第一要务，着力扩总量、调结构、惠民生，求真务实，开拓进取，圆满完成了市五届人大五次会议确定的各项目标任务，在推进富民强市的道路上迈出了坚实步伐。

　　（一）国民经济持续快速健康发展。地区生产总值完成1347.2亿元，增长15.5%，高于全区1.2个百分点，增速居全区第3位。三次产业协调发展，第一产业增长7.1%，第二产业增长20.4%，第三产业增长12%。投资、消费稳步增长，全社会固定资产投资1115.1亿元，增长31.4%，高于全区9.9个百分点；社会消费品零售总额399.9亿元，增长18.8%，高于全区0.8个百分点。经济增长的质量和效益明显提高，地方财政总收入120.6亿元，增长20%，收入结构进一步优化，地方财政总支出272亿元，居全区第2位，增长23.8%；规模以上工业增加值增长22%，高于全区3个百分点，增速居全区第3位。全市经济实现了速度、质量、效益协调同步、快速增长。

　　（二）经济结构调整取得积极进展。农牧业1571工程成效明显。完成玉米膜下滴灌108万亩、高效节水经济林22万亩、节水人工种植紫花苜蓿12.7万亩，设施农业总面积达到71万亩；综合生产能力大幅提升，粮食总产达到87亿斤，比上年增加16亿斤；牧业年度家畜存栏1720万头只，连续六年保持全区第一。工业"双千双百亿"工程进展顺利。金剑铜业、远联钢铁年销售收入双双突破百亿元大关；机械制造、化学工业分别增长88.1%和48.8%，对规模以上工业增长的贡献率提高了5.5个百分点，达到16.5%；实施3000万元以上工业项目257个，完成投资355亿元，中色四期、国电煤制尿素、金剑电解铜、远联三期等一批大项目竣工投产。现代服务业加速发展。红山物流园区实现交易额150亿元，晋升为自治区现代化综合物流园区，集聚辐射作用显著增强；商贸流通业重点项目年度投资首次突破百亿元大关，实现翻番增长，23个投资超亿元的大型商贸项目启动建设，新增建筑面积300万平方米；全市接待游客640万人次，实现旅游收入88亿元，观光、休闲、体验、文化等特色旅游业蓬勃发展；新增各类金融机构16家，金融机构存款余额1037亿元，增长17.3%，贷款余额565亿元，增长23%，企业上市工作和要素市场建设扎实推进。

　　（三）人民生活持续改善。城镇居民人均可支配收入16416元，农牧民人均纯收入6138元，分别增长16.4%和22.5%，增速居全区第1位和第2位，增幅为近年来最高。财政对民生领域的投入占财政总支出的46.5%。新增城镇就业2.36万人，落实应往届高校毕业生就业1.39万人，失业人员再就业1.29万人，农牧民转移就业70.8万人。建立企业职工工资正常增长机制，增加行政事业单位人员津补贴，较大幅度地提高了企业退休人员养老金、城乡低保、五保供养标准。全面落实国家和自治区惠农惠牧政策，发放粮食直补、农牧业生产资料综合补贴、农作物良种补贴和农机具购置补贴9.36亿元、草原生态奖补资金4.31亿元。扶持贫困人口9万余人，解决了农村牧区36万人的饮水安全问题。新农保试点范围扩大到6个旗县，城乡居民养老保险制度更加完善，更多群众从中受益。出台《赤峰市老年人优惠优待暂行规定》并在全区推广，老年人享受了免费乘坐公交车、免景区首道门票费、免普通挂号费等优待政策。一批综合福利中心项目投入使用，社会福利水平进一步提升。

　　（四）社会事业全面进步。农博会升格为国家级展会，被科技部命名为中国北方农业科技成果博览会。新增国家级高新技术企业4家，内蒙古蒙药产业技术创新联盟落户我市，昭乌达肉羊新品种通过农业部验收命名。校安工程三年规划任务基本完成，竣工率居全区前列。学前教育三年行动计划正式启动，双高普九各项指标大幅提高，高中阶段免费教育惠及7.2万名学生，高考本科上线率高出自治区10.3个百分点。松山区职教中心成为国家示范中等职业学校，内蒙古纺织工业学校升格为赤峰工业职业技术学院，赤峰学院新建本科院校合格评估工作进展顺利。新建改造市县两级医院和基层卫生服务机构189个，疾病防控救治能力进一步提高，全市医疗卫生服务体系日趋健全。成功举办了第六届红山文化节。新建苏木乡镇综合文化站和草原书屋723个，各级公共文化场馆全部免费向公众开放，覆盖城乡的公共文化服务体系进一步完善。重点文化遗产保护加快实施，辽代大遗址群列入自治区申遗名单，力王公司被文化部命名为国家文化产业示范基地。人口和计划生育工作健康发展，低生育水平保持稳定。第六次人口普查工作圆满完成。全区第十三届运动会筹备工作进展顺利。人防、气象、地震、档案、慈善、红十字、外事、侨务、残疾人、妇女儿童等工作都取得新成绩。

　　（五）可持续发展能力进一步增强。城镇化水平稳步提高。全市城镇化率提高1.5个百分点，达到44.7%。中心城区改老城、扩新区、修环路、保安居工程进展顺利，老城区百万平米老旧小区改造基本完成，1.3万户居民从中受益；桥北组团一期主干路网基本建成，1300户居民回迁入住，松北新城、小新地组团建设全面展开；环城高速公路建成并实现免费通行，中环路启动建设；开工建设保障性住房3.45万套，超额完成自治区下达任务。中心城区环卫、绿化、亮化、美化水平进一步提高，顺利通过了国家卫生城复审。旗县城关镇开发建设步伐加快，重点小城镇承载和集聚能力进一步增强。生态建设和环境保护成效明显。重点生态工程完成投资14亿元，完成营造林321.4万亩，治理水土流失106.4万亩，草原禁牧和草畜平衡面积8539万亩；国家森林城市创建工作取得阶段性成果；重点区域、流域和重金属污染治理力度加大，淘汰了一批资源消耗大、环境污染重的落后产能。基础设施进一步改善。交通建设完成投资61.2亿元，赤朝高速公路建成通车，赤承高速公路、赤凌一级路建设加快，又有124个嘎查村通油路；铁路在建规模472公里，赤锦铁路、林白铁路进展较快；民航航线增加到9条，客运量首次突破30万人次；水利、电力建设完成投资25.8亿元，一批农村牧区水利工程及输变电项目投入使用。

　　（六）改革开放扎实推进。企业改革迈出新步伐，兴业集团成功重组富龙热电。集体林权主体改革基本完成，累计确权4206万亩，占应改面积的99.4%。财政奖补工作成效显著，全市实施一事一议项目1225项，总投资5.99亿元，均居全区首位。启动了公立医院改革试点工作，完成基层医药卫生体制综合改革任务，引进国内区外资金625.6亿元，实际利用外资3675万美元。对外贸易快速增长，进出口总额达到12.2亿美元，增长15%。

　　（七）民主法制建设全面加强。全年向人大常委会报告工作14次，向政协常委会通报情况6次，人大代表建议、意见和政协委员提案办结率100%。水资源保护和管理、中小企业发展、校安工程等事关经济社会发展的重大事项被纳入人大和政协监督工作的重要议题，有力促进了政府工作。深入开展民族团结进步和群众性精神文明创建活动，启动全国文明城市创建工作并进入提名城市行列。实施六五普法规划，加大行政监察和审计监督力度，对重点领域的突出问题实施专项治理，政府机关廉政建设进一步加强，依法行政工作扎实推进。加强和创新社会管理，阿旗村级事务契约化管理经验在全区推广，社区建设管理水平不断提高，全市改扩建社区办公和活动场所62个，新增自治区级和谐社区8个。开展信访积案化解百日攻坚专项行动，一大批涉及群众切身利益的问题得到解决，信访总量大幅下降。食品药品安全监管得到加强，重点领域安全生产形势总体稳定。完善社会应急管理体系，处置突发公共事件的能力进一步增强。深入开展严打整治行动，依法打击各类犯罪活动，人民群众的安全感和满意度不断增强。积极支持国防动员和后备力量建设，军地共建再创佳绩，实现了全国双拥模范城七连冠。

　　各位代表，过去一年取得的可喜成绩，是市委正确领导、几大班子通力合作的结果，是全市各族干部群众团结一心、共同奋斗的结果，也是社会各界建言献策、鼎力相助的结果。在此，我代表市人民政府，向全市各族人民，向在各条战线上辛勤工作、付出努力的同志们，向所有关心和支持赤峰发展的朋友们，致以崇高的敬意和衷心的感谢！

　　在看到成绩的同时，我们也清醒地认识到，我市欠发达的基本市情还没有根本改变，经济社会发展中仍存在不少困难和问题。主要是：调结构、转方式的任务比较繁重。经济总量小，结构性矛盾比较突出，产业发展层次不高，保持全市经济持续快速增长压力较大；生态、环保和城乡基础设施建设力度需进一步加大。乡村道路、能源运输通道建设仍是薄弱环节，节能减排、水资源保护和矿业秩序整顿任务艰巨；民生和社会保障能力薄弱。就业、扶贫和城乡居民持续增收仍需攻坚克难，涉及群众切身利益的问题有待进一步解决；社会建设和管理需进一步加强。基本公共服务水平不高，社会事业发展相对滞后，社会管理创新还有大量工作要做；政府自身建设还需不断强化。政府职能转变与科学发展要求不相适应，服务意识、服务能力、服务水平亟待提高，廉政建设和机关作风建设需进一步改进。对这些困难和问题，我们将高度重视，并在今后的工作中认真加以解决。

　　二、2012年主要工作

　　今年是深入实施十二五规划承上启下的重要一年，做好今年的工作，意义重大。

　　今年政府工作的总体思路是：坚持以科学发展观统揽全局，全面贯彻中央和自治区经济工作会议、自治区第九次党代会和市第六次党代会精神，牢牢抓住中央促进内蒙古又好又快发展的重大机遇，努力推动科学发展，加快富民强市进程，全力保持经济持续快速增长，加快推进经济结构调整和发展方式转变，提高科技创新能力，着力加强生态、水资源保护和基础设施建设，不断深化改革，扩大开放，切实保障和改善民生，全面推进精神文明、民主法制和政府自身建设，保持社会和谐稳定，以经济社会又好又快发展的优异成绩迎接党的十八大胜利召开。

　　主要预期目标是：全市生产总值增长17%左右；地方财政总收入增长20%；全社会固定资产投资增长21%；社会消费品零售总额增长18%；城镇居民人均可支配收入和农牧民人均纯收入分别增长15%；完成自治区下达的各项节能减排任务。

　　上述目标，总体上略高于自治区年度目标和全市十二五规划目标，体现了加快发展、科学发展的主题。总的要求是，保增长、调结构、增投资、强基础、惠民生、促和谐。在具体工作中，必须把握好以下原则。一是紧抓发展第一要务不动摇。坚定不移地做大经济总量，坚定不移地调整经济结构，坚定不移地转变发展方式，把经济增长建立在结构优化、能耗降低、质量提升的基础之上，提高经济发展的整体竞争力和抗风险能力。二是强化项目第一抓手不动摇。始终把项目建设作为实现目标的主要载体、落实任务的直接抓手、检验工作的重要标尺，一个项目一个项目地抓落实、求突破、见成效。三是突出民生第一目标不动摇。把扶贫攻坚作为头号民生工程，落实一把手责任，全力打好新一轮扶贫开发攻坚战，继续增加民生投入，办好惠民实事，努力推动经济发展与就业增加、群众增收相协调，进一步提高城乡居民生活水平，促进城乡基本公共服务均等化。四是履行维稳第一责任不动摇。更加注重基层防控体系建设，更加注重满足群众合理诉求，更加注重依法行政，巩固和发展各族人民团结奋进的大好局面，推动和谐赤峰建设再上新水平。

　　今年重点要抓好八个方面的工作：

　　（一）推进产业优化升级，提高工业经济质量和效益

　　扎实推进双千双百亿工程。按照打造大产业、培育大企业、建设大园区的思路，进一步做大做强工业经济。推动中色14万吨锌冶炼、远联100万吨优质线材等项目开工建设，山金10万吨铅冶炼、半拉山铜钼采选等项目建成投产，力促远联三期、富邦铜冶炼等项目达产达效，推动冶金产业率先成为销售收入突破千亿元的产业。引导鼓励大企业大集团对我市企业兼并重组、注资入股，实现合作发展；支持现有企业扩体增量、提档升级，壮大骨干企业实力，推动平庄煤业等企业成为新的百亿元企业。加大工业园区基础设施投入，优化产业布局，推动园区扩容升级，加快项目引进和建设进度，促进产业集聚发展；抓好化工、食品、机械制造、有色金属深加工、建材、循环经济等专业园区建设，培育经济增长的新优势；全市工业园区销售收入突破800亿元，力促玉龙工业园区、宁城经济开发区进入百亿元园区行列。

　　促进产业优化升级。一手抓好资源型产业延伸升级、提质增效，一手抓好非资源型产业培育扶植、增量扩张，进一步优化产业结构，促进多元发展。提升有色金属精深加工水平，抓好亚鼎铜材、京铜异型铜、金奥电工、华润铝业等项目，延长有色金属产业链条；扩大煤化工、氟化工、玉米化工规模，确保大唐煤制气一期投产，启动国电煤制尿素二期工程，抓好伊品氨基酸、国能焦油加氢、天一氟化工等项目建设；促进煤电一体化发展，开工建设中电投2×30万千瓦热电联产项目，实现大板电厂并网发电，抓好京能二期2×35万千瓦、克旗2×66万千瓦发电机组前期工作；保持绿色能源发展强势，建设阿旗罕山、克旗骆驼台子等大型风电项目，新增风电装机50万千瓦以上；推动食品工业加快发展，抓好红山农畜产品加工产业园、塞飞亚食品工业园建设，力促雨润牛羊屠宰、吉敖肉鸡加工等食品工业项目建成投产；加快非金属矿产资源开发利用，科学规划设置矿业权，寻求技术合作，选择战略投资者，新建一批非金属资源开发项目；加大探矿增储，提高矿产资源整合利用水平。推动机械制造、电子信息等非资源型产业快速扩张，促进国电风机、北奔重汽、金成重工、鸿宇精密等企业加快发展，抓好风机总装、重型装备、矿山设备制造、汽车零部件加工及五甲万京、三和电子等项目建设，构建多极支撑的产业发展格局。

　　全力支持中小企业发展。实施中小企业成长工程，落实国家和自治区促进中小企业、微型企业发展的政策措施，优化市场准入、融资支持、土地供给等方面的发展环境。加强中小企业创业园区建设，扶持和引导中小企业围绕优势特色产业搞延伸，围绕重点项目搞协作，培育特色产业集群。加快中小企业诚信体系和服务体系建设，落实中小企业信贷政策，积极拓展中小企业创业投资和产业投资基金等直接融资渠道。重视并加强企业家队伍建设，推动更多的本土企业发展壮大。

　　（二）加强农牧业基础建设，提高现代农牧业发展水平

　　大力推进节水高效农牧业。紧紧依靠科技兴农兴牧，不断优化农牧业发展结构，提高综合生产能力。围绕百亿斤粮食生产能力建设，加强农业综合开发和土地整治，提高粮食单产水平，粮食播种面积稳定在1500万亩以上。积极推广喷灌、滴灌等高效节水灌溉技术，新增玉米膜下滴灌100万亩、高效节水经济林20万亩、设施农业10万亩，建设北方高效节水农业示范区。加快以苜蓿种植为重点的高效节水饲草料基地建设，完成高效节水苜蓿种植35万亩，到"十二五"末达到200万亩，在蒙东地区率先建成苜蓿优势产区。全年家畜存栏达到1750万头只以上，出栏优质育肥牛90万头以上。加快推广昭乌达肉羊品牌，扩大养殖规模，提高经济效益。

　　提升产业化发展水平。加强农牧业产业化园区和基地建设，努力在粮、肉、菜、草、乳及生态产业上引进培育一批新的龙头企业，增强辐射带动能力。支持雨润、亚盛、温氏集团等大型龙头企业在我市整合资源、建设基地、发展精深加工项目，支持塞飞亚、蒙都羊业、大牧场等龙头企业做大做强。扎实推进农畜产品标准化生产、市场监测预警、质量安全监管、重大动植物疫病防控等服务体系建设，积极发展绿色有机农畜产品，打造区域特色品牌。大力发展农牧民专业合作组织，鼓励发展种养大户和家庭农牧场，扩大订单农牧业规模，提高农牧业生产经营的组织化水平和市场开拓能力。

　　抓好扶贫攻坚和农村牧区基础设施建设。建立各级财政扶贫投入稳定增长的长效机制，全力实施好连片特困地区扶贫、整村推进扶贫、易地移民搬迁扶贫、产业化扶贫、社会扶贫五大工程，扶持8.5万人实现稳定脱贫。大力推进农田水利建设，继续实施大中型灌区续建配套工程，完成哈日朝鲁等7座病险水库除险加固，改造中低产田13.5万亩。积极发展民生水利，实施饮水安全工程，解决30万人饮水安全问题。搞好中小河流治理和山洪地质灾害防治。加快推进乡村道路、供水供气、电力通讯等基础设施建设，新建通村油路1000公里。

　　（三）推动服务业提档升级，提高第三产业发展水平

　　大力发展商贸物流业。着力提升传统商贸业层次和水平。抓好中心城区维多利购物中心、天丰商贸城及印象红山、新天地、永业城市广场等大型商贸项目建设，开工建设万达广场，引进王府井等知名品牌；加快旗县大型商场、高档宾馆和娱乐服务项目建设。积极发展现代物流业。立足于培育大市场、大物流，调整完善物流业发展规划，统筹推进红山物流园区、松山物流园区、和美工贸园区建设和发展，在中心城区近郊建设大规模、多业态、高层次的物流产业带。抓好蒙东云计算中心、国际陆港、保税物流中心等高端业态项目建设，推动雨润农副产品交易配送中心、安徽商会农资市场开工建设，启动五金机电城二期工程，提高物流配货能力，培育市场品牌；抓好大板、敖汉、乌丹、上京等物流园区和专业市场建设，着力打造煤炭、粮食、蔬菜等物流集散地。加快培育大型现代物流企业，推广应用先进物流技术，实现物流业在较高层次上的快速发展。

　　推动旅游业与文化产业融合发展。深度挖掘旅游业的文化内涵，充分发挥文化促进旅游消费的作用，提升旅游业发展层次。依托石林、草原、森林、湖泊、冰臼、冰雪等优势旅游资源，开发特色旅游项目，完善旅游设施，做足夏秋旅游，搞活冬季旅游，集中打造克什克腾旅游品牌，全面提升知名度和美誉度。大力开发温泉旅游资源，建设集养生、休闲、观光、商务于一体的温泉旅游度假基地。规划、开发赤峰至茅荆坝百公里文化旅游产业带，推动南部旗县区旅游经济加快发展。抓好重点旅游景区基础设施和接待能力建设，打造一批精品、特色旅游景区，推动乌兰布统、美林谷、玉龙沙湖、真寂之寺等景区提档升级，加快国家5A级旅游景区创建步伐。推进与曲江集团、中旅集团等知名文化旅游企业及周边盟市的合作，促进优势资源集约开发。筹建文化产业投融资平台，抓好辽上京文化产业园、和美文化产业园建设，启动实施赤峰大剧院、中国印城项目。加快赤峰旅游综合服务中心建设，加强旅游市场管理，提升旅游业服务水平。强化旅游宣传，深化与央视等传媒的合作。大力开拓旅游客源市场，全年接待游客720万人次，实现旅游收入110亿元。

　　健全完善金融服务体系。新增小额贷款公司10家、村镇银行2家、驻赤商业银行旗县分支机构5家，推动元宝山农合行、松山信用联社改组改制为农村商业银行，加快引进外埠金融机构，增强金融业服务经济社会发展的能力。加大对三农三牧、承接产业转移、中小企业、民生工程的信贷支持力度，信贷投放总额增长20%以上。规划建设金融商务区，打造集聚发展、服务全市的金融产业区。推进中小企业集合债券、票据发行工作，发展有色金属、新能源、绿色农畜产品私募基金，培育债券融资和股权投资市场。继续做好企业上市工作，推动塞飞亚、凌志马铃薯、吉隆矿业等企业尽快进入资本市场。

　　扩大城乡居民消费。坚持以增收促消费，提升城乡居民消费能力。加快培育新的消费热点，推动消费结构升级。健全社区服务网络，大力发展家政、养老、托幼等便民服务业。拓展休闲、健身、通讯等消费领域，促进科技、教育、文化等精神产品消费。推进新网工程、万村千乡市场工程和家电下乡工程，新建、改造配送中心10个、农村牧区各类经营网点320个，新增标准化农家店150家。加强鲜活农畜产品流通体系建设，切实保障市场供应。认真落实稳控物价的各项政策措施，大力整顿和规范市场秩序，全面优化消费环境。

　　（四）加强基础设施建设，提高可持续发展能力

　　加快推进城镇化进程。坚持规划先行，完成赤峰市城市总体规划修编，启动中心城区供热、供水、供气、交通、绿地、公厕和西山公园等专项规划编制，修编小新地组团、八家组团控制性详细规划，提高旗县城镇规划编制水平。加大城镇基础设施建设力度，全市计划实施市政建设项目157个，完成投资76亿元。继续推进改老城、扩新区、修环路、保安居工程，全面提升中心城区建设水平。完成老旧小区改造100万平方米，有序实施棚户区、城中村改造，新建各类保障性住房2.3万套。进一步完善八家组团服务功能，推进小新地组团建设。加快桥北组团、松北新城基础设施、专业小区和商业开发等建设，继续拉大城市框架，增强中心城区承载辐射能力。理顺新区管理体制，加大统一规划、监管、协调服务力度，促进组团之间基础设施对接和产业协调发展。规范和提升出租车服务水平，大力发展城市公交，改造建设一环两路，提高中心城区通行能力。加强旗县城关镇和重点小城镇建设，推动基本公共服务向基层延伸，加快城乡一体化进程。打造建筑业品牌优势，整合资源，提升层次，进一步提高建筑业对经济增长的贡献率。

　　加强生态建设和环境保护。继续实施京津风沙源治理、三北防护林等重点工程，完成营造林300万亩，科学规划并启动建设1500万亩樟子松基地。加强湿地生态保护，力争将我市重点湿地列入国家保护规划，争取更多的扶持。全面完成国家森林城市创建任务，确保通过国家验收，为赤峰再添一张国家级城市名片。落实好草原生态奖补政策，推进封育禁牧、压减草畜平衡区放养牲畜、生态移民等重点工作，实现草原生态的进一步好转。大力发展林沙草产业，促进林区、牧区群众增收。实行最严格的水资源管理制度，强化全社会节水意识。实施地下水保护行动，启动三座店水库向中心城区引水、中心城区10万吨污水处理扩建等工程，鼓励和推动中心城区工业企业使用中水，严禁开采地下水新建高耗水工业项目。认真抓好节能减排工作，推动工业、交通、建筑、公共机构等各领域全面节能，推进主要污染物总量减排，强化环境风险防范和重金属污染防治，推广使用清洁能源。抓好大气环境综合整治工作，集中清理中心城区烟尘粉尘排放污染源。

　　加快交通能源通道建设。推进大板至林西一级公路前期工作，开工建设新惠至萨力巴一级公路，确保赤凌一级公路主体完工，赤承高速公路铺筑面层。做好京沈客专连接线、京通铁路扩能改造、赤大白增二线前期工作，确保在建和已核准铁路项目进度。推进玉龙机场扩建工程，增加航班航线，适时启动机场迁建前期工作。完成赤峰至北京天然气管道建设，实现向北京供气。实施"气化赤峰"工程，完成朝阳至赤峰城区天然气输气管道建设，启动克旗至赤峰城区及贯通各旗县区的天然气输气管道建设，力争年内实现南部旗县区的贯通。开工建设220千伏汐子输变电工程和赤一变、城东变扩建工程，确保220千伏经西、绍根输变电工程投入运行，提高电力保障能力。

　　（五）继续深化改革开放，增强发展活力和动力

　　推进重点领域改革。深化农村牧区综合改革，推动土地和草牧场合法有序流转，积极发展多种形式的适度规模经营。扩大嘎查村级公益事业一事一议财政奖补覆盖面，在旗县区开展公务卡试点。加快集体林权制度配套改革，开展旗县林权管理中心建设示范工作。推进行政审批制度改革，清理和规范行政审批事项，抓好市行政审批服务中心迁建工作。进一步清理涉及企业的行政事业和经营服务收费，减轻企业负担。稳步推进事业单位人事聘用和绩效工资改革。

　　提高对外开放水平。采取更加灵活、开放的政策和积极可行的措施，进一步优化投资环境，扩大区域合作领域，提升区域合作水平。积极落实京蒙对口帮扶框架协议，扩大与北京市的全方位合作。继续面向环渤海、东北、长三角和珠三角等地区搞好招商推介，积极参与内蒙古香港经贸周活动，力争在承接产业转移、与周边地区基础设施对接、产业带共建等方面取得新的突破。通过招商同步、资源共享、指标分计、利益分成等灵活方式，积极发展飞地经济，推动与周边地区水煤合作取得实质性进展。支持东黎、维信、华源等企业优化出口结构，开拓外贸市场，完成进出口总额14亿美元，增长15%以上。

　　（六）切实保障和改善民生，全面发展社会事业

　　千方百计增加城乡居民收入。积极拓展城乡居民增收渠道，努力实现居民收入增长与经济增长同步。认真落实规范公务员津补贴、推进事业单位绩效工资制度改革的各项政策措施，建立健全企业职工工资正常增长和支付保障机制。采取切实有效措施，促进农牧民持续增收。通过提高农牧业综合生产能力，发挥农村牧区合作经济组织带动作用，完善产业化利益联接和农牧民与市场对接机制，增加经营性收入；加快发展农村牧区二三产业，加强对农牧民转移就业的培训，以就业促增收，增加工资性收入；完善征地、拆迁、草场征用等补偿措施，引导农牧民以土地、草场、林权等参与收益分配，增加财产性收入。继续加大投入，全力办好事关群众当前和长远利益的实事、好事，推动民生工作向保基本、广覆盖、纵深化方向发展。

　　全力做好就业工作。全面落实促进就业的各项政策，完善职业技能培训、劳务对接、人才储备等服务措施，推进就业服务实名制管理。加强就业信息服务平台建设，促进人力资源供需对接。充分发挥企业吸纳就业主渠道作用，支持鼓励更多的高校毕业生、下岗职工到企业就业，努力开发基层社会管理和公共服务岗位，新增城镇就业2.7万人。实现农牧民转移就业71万人。大力扶持城乡居民自主创业，设立创业发展资金1500万元，建立创业孵化基地13个，力争进入国家级创业型城市行列。

　　完善社会保障体系。推进覆盖城乡居民的社会保障体系建设，实现新型农村牧区社会养老保险和城镇居民养老保险制度全覆盖。扩大失业、工伤和生育保险覆盖范围。增强基本医疗保险保障能力，实行新农合市级统筹，推进城镇职工基本医疗保险、新农合与商业保险的经办合作，住院报销封顶线均提高到20万元。扩大城镇居民医疗保险一卡通实施范围，实行定点医疗机构总额预付制度。推动救灾物资储备体系建设，提高防灾救灾保障能力。落实社会救助与物价上涨挂钩联动机制，完善城乡低保和临时救助制度，加强社会救助中心基础建设，提高优抚对象补助标准，推进农村牧区敬老院法人登记工作，发展慈善事业。完成赤峰市军休服务中心项目建设，筹建赤峰市老年护理院和残疾人康复中心。足额发放高龄津贴，落实孤儿保障政策，加强流浪乞讨人员救助管理。规范保障性住房建设、分配和管理，建立和完善多层次、多渠道住房保障体系，逐步解决低收入家庭住房困难问题。

　　坚持优先发展教育。推进基础教育内涵发展，进一步强化素质教育，深化新课程改革，全面提高教育教学质量。加快实施学前教育三年行动计划，新改扩建公办幼儿园133所。启动实施2012-2015年校舍加固工程，完成投资2.7亿元，加固校舍34万平方米。巩固提高义务教育水平，提前一年实现高中阶段免费教育。优先重点发展民族教育，大力发展职业教育，新创建国家级中等职业教育示范校1所。加快赤峰工业职业教育集团组建步伐，加强内蒙古交通职业技术学院基础能力建设，支持赤峰学院打造科技文化创新基地和区域性人才高地。

　　加强科技人才工作。推进科技基础设施、创新平台和创新载体建设，加快建立以企业为主体、市场为导向、产学研相结合的技术创新体系。加快科技成果转化和产业化步伐，加大科技投入，提高科技创新对调结构、转方式的支撑和保障能力。办好中国北方农业科技成果博览会，打造具有较高知名度和影响力的展会品牌。加快赤峰国家农业科技园区建设步伐，积极培育有色金属产品开发、农机制造、草业技术创新战略联盟，实施各类科技攻关计划。扎实推进玉龙人才工程，设立专项资金用于人才引进和培养，依托京蒙人才交流合作等平台，积极引进各类优秀人才。

　　加快医药卫生事业发展。继续深化医药卫生体制综合改革，巩固政府办基层医疗卫生机构综合改革和国家基本药物制度建设成果，加强基层医疗卫生体系建设，抓好市级卫生应急救援指挥中心、8个卫生监督所、12个妇幼保健所等项目建设，推动在建医院尽快投入使用，进一步提高医疗卫生服务水平。推进县级公立医院改革，建立和完善法人治理机制、运行机制和投入补偿机制。启动实施新一轮京赤卫生对口支援与技术协作规划，扩大协作领域和范围。积极发展蒙医中医事业。认真做好人口和计生工作，深入开展整治两非专项行动，提高出生人口素质，稳定低生育水平。大力开展爱国卫生运动，巩固国家卫生城创建成果。

　　发展文化体育事业。加强社会主义核心价值体系建设，深入开展全国文明城市创建活动，不断提高全社会的道德文明水平。通过全民参与，提炼新时期赤峰精神，使之成为全市人民认同并践行的行为准则。发展文化事业，推进文化体制改革。深入发掘和弘扬民族文化，大力开展面向群众、贴近生活、反映时代的文学艺术创作，努力推出一批具有较强影响力和传播力的精品力作。继续实施文化惠民工程，建设苏木乡镇综合文化站75个、草原书屋318个，实现嘎查村草原书屋全覆盖。办好第七届红山文化节等系列节庆活动，打造节庆品牌。深入开展文化遗产保护、发掘和利用工作，筹建二道井子遗址博物馆，启动红山遗址群申遗工作，力争年内辽代大遗址群进入国家申报世界文化遗产预备名单。大力推进广播影视数字化进程，抓好村村通工程，推动蒙古语专业频道尽快开播。加强与周边及发达地区的文化交流与合作，发挥文化在与国内外友好城市交往中的纽带作用，提升具有赤峰特色的草原文化影响力和知名度。落实全民健身计划，积极开展群众性体育活动，加快体育中心建设，认真做好全区第十三届运动会、全市第二届运动会的筹备工作。加强统计、档案、人防、气象、红十字、外事、侨务、地震等工作，维护妇女儿童、老年人和残疾人合法权益。

　　（七）加强社会管理，维护社会稳定

　　推进社会管理创新。加强基层基础建设，抓好社会管理创新综合试点工作。整合苏木乡镇、城乡社区的社会管理资源，积极推进网格化管理、精细化服务模式，提升社区管理服务水平，社区办公和活动场所面积全部达到300平方米以上。平稳有序完成新增苏木乡镇设立和第八次嘎查村委会换届工作，深入推进嘎查村级事务契约化、社区化管理，提高群众自我管理、自我发展能力。积极引导和发展社会中介、行业协会、公益慈善等社会组织。加强非公有制经济组织和新社会组织规范化建设，强化企事业单位社会服务和管理责任。加强六五普法宣传，深入开展平安创建工作，提高全民法制意识和全社会法治化管理水平。强化法律援助，完善人民调解、行政调解、司法调解与仲裁相衔接的大调解体系。完善社会治安防控体系，依法打击各种违法犯罪活动，提高预防和应对突发公共事件的能力。全面贯彻党的民族宗教政策，依法管理民族宗教事务。支持驻赤部队建设，加强民兵和预备役建设，做好双拥工作，增进军政军民团结。

　　加大信访工作力度。坚持以群众工作统揽信访工作全局，扎实推进信访工作基层基础建设和体制机制创新。落实信访工作领导责任制，深入开展领导干部大接访、大走访活动，认真解决群众反映的热点难点问题。进一步加大社会矛盾纠纷排查化解力度，引导群众依法、逐级、有序表达诉求，积极预防和妥善处置群体性信访事件，集中清理、解决信访积案，确保进京非访稳定退出全区前四、赴区非访退出前五。全面落实社会稳定风险评估机制，突出抓好前置准入、决策评估和事后问责三个环节，推动社会矛盾防控和化解工作规范化、常态化。

　　抓好安全生产和食品药品安全工作。进一步完善安全生产管理体制和联合执法工作机制，落实企业安全生产主体责任和部门监管责任，加强对矿山、化工、交通、建筑、消防等高危行业和重点领域的安全监管，切实抓好校车安全，坚决遏制重特大事故发生。理顺食品药品安全监管体制，进一步强化监管职能，推行基本药物电子监管，逐步建立食品药品安全监管的长效机制。抓好各领域专项治理，全力保障人民群众生命健康和财产安全。

　　（八）加强政府自身建设，努力打造务实高效廉洁政府

　　完成今年的目标任务，各级政府责任重大。要切实加强政府自身建设，努力做到对人民负责、受人民监督、为人民造福。

　　严格依法行政。把依法行政作为政府工作的基本准则，坚持用制度管权、按制度办事、靠制度管人，严格按照法定权限和程序行使权力。自觉接受人大的法律监督、工作监督和政协的民主监督，认真办理人大代表议案、建议和政协委员提案。充分听取民主党派、工商联、无党派人士及各人民团体的意见，接受新闻舆论和社会公众监督。健全集体决策、专家咨询、社会公示与听证等制度，促进民主决策、科学决策。改进执法方式，规范行政执法，严格落实执法责任制，营造良好的政务环境。

　　坚持执政为民。始终把执政为民作为政府工作的出发点和落脚点，更加注重人文关怀，更加注重社会管理和公共服务，做到问政于民、问需于民、问计于民，使提出的发展思路、工作部署、政策措施更加符合实际、更加贴近民愿，使更多的资源向推动发展倾斜，更多的财力向改善民生倾斜，多做群众急需、群众受益的事，多做打基础、管长远的事，真正让人民群众共享改革发展成果。

　　狠抓工作落实。强化政府绩效管理，层层分解目标任务，完善跟踪问效、考核问责的督查机制，构建一级抓一级、一级对一级负责的目标责任体系。坚持从政府一班人做起，大力倡行深入一线的工作作风，脚踏实地，少说多做，对重大决策部署、重点工作安排说了不忘，盯住不放，一抓到底，抓出成效。大力精简会议、文件，控制考察、接待和机关公用经费支出。严厉查处不作为、乱作为和有令不行、有禁不止、失职渎职行为。

　　加强廉政建设。抓好政府投资和国有资产项目的评审、管理、监察和审计，提高资金使用效益。加强工程建设领域和商业贿赂专项治理，加强对工程招投标、土地出让、城建规划、产权交易、资源开发、政府采购等重点工作的监督管理，加强救灾资金、社保资金、住房公积金、扶贫资金等重要资金的审计监管，严肃查处违法违纪行为，树立清正廉洁的政府形象。

　　各位代表，科学发展的目标鼓舞我们团结奋进，富民强市的重任激发我们勇于担当。让我们在市委的正确领导下，紧紧依靠全市各族人民，坚定信心，奋力拼搏，乘势而上，不断开创各项工作的新局面，谱写赤峰发展振兴的新篇章！
